# Role of Ion Channels in the Development of Pulmonary Arterial Hypertension

**DOI:** 10.3390/biom12101373

**Published:** 2022-09-25

**Authors:** Fabrice Antigny

**Affiliations:** INSERM UMR_S 999 «Hypertension Pulmonaire: Physiopathologie et Innovation Thérapeutique», Hôpital Marie Lannelongue, 133, Avenue de la Résistance, F-92350 Le Plessis Robinson, France; fabrice.antigny@inserm.fr; Tel.: +33-1-40-94-22-99

Pulmonary arterial hypertension (PAH) is an uncommon, progressive, and fatal disease. Under the current clinical classification, PAH consists of different etiologies leading to precapillary pulmonary hypertension (PH), which is hemodynamically defined by a mean pulmonary artery pressure (PAP) >20 mmHg, pulmonary artery wedge pressure <15 mmHg, and pulmonary vascular resistance (PVR) >2 Wood units at rest [1]. PAH is termed idiopathic (IPAH) when no causative factors are identified. However, PAH can also be heritable (hPAH), induced by drugs or toxins, or associated with other pathologies [1,2]. PAH results from increased PVR due to narrowing of the small distal pulmonary arteries (diameter < 500 µm), causing adaptive right ventricular hypertrophy and right heart failure [1]. No cure exists for PAH, and mortality after three years remains high, at 30–40%, despite the available therapies, mainly consisting of vasodilators targeting endothelial dysfunction [2]. These therapies approved in treating PAH act against three dysfunctional pathways: endothelin, nitric oxide, and prostacyclin. Novel therapeutic hypotheses that target pulmonary vascular remodeling are urgently needed to advance PAH management. 

The recent identification of mutations in genes encoding for ion channels, *KCNK3*, *ATP13A3*, and *ABCC8,* revived the interest in ion channels in the pathological context of PAH [3,4,5]. PAH is a multifactorial and complex disease characterized by pulmonary arterial (PA) smooth muscle cell (PASMC) and endothelial cell (PAEC) dysfunctions, leading to excessive PA vasoconstriction, exacerbated proliferation of PASMCs and PAECs, endothelial–mesenchymal transition, and apoptosis resistance. These phenomena could be driven by a remodeling of ion channels, including K^+^, Ca^2+^, and Cl^−^ channels. Indeed, many ion channels are described to regulate the functions of pulmonary vascular cell phenotypes. 

This Special Issue of *Biomolecules*, entitled “Role of Ion Channels Signaling Pathways in the Development of Pulmonary Arterial Hypertension,” provides novel findings regarding ion channels in PAH and an updated overview of the main aspects of ion channel remodeling in PAH pathogenesis. Eight manuscripts, two original research articles, and six reviews encompass several aspects of ion channel signaling in the pulmonary vasculature.

In their review, Joana Santos-Gomes and collaborators focused on the potential pathogenic role of different ion channel families (K^+^, Ca^2+^, Na^+^, and Cl^−^ channels) in the initiation and progression of endothelial dysfunction in PAH pathogenesis and their potential therapeutic interests. Endothelial dysfunction plays a critical role in PAH; therefore, a better understanding of the endothelial dysfunction PAH should facilitate the evolution of newer, targeted therapy. This review highlights that most ion channel families are functionally expressed in healthy PAECs. However, it highlights the need for more studies to decipher the role of ion channels on endothelial dysfunction observed in PAH [6]. 

Most functions of such pulmonary vascular cells, including the contraction, migration, proliferation, and production of extracellular matrix proteins, are regulated or controlled by variations in intracellular calcium concentration ([Ca^2+^]i), consequences of an influx from the extracellular compartment, and/or a release of stored intracellular Ca^2+^. The paper by Bastien Masson and colleagues reviewed the current knowledge in Ca^2+^ entry, called store-operated Ca^2+^ entry (SOCE) in PASMCs. SOCE is selective or non-selective; Ca^2+^ channels constituting Orai channels and TRPC channels (transient receptor potential canonical) are activated after intracellular Ca^2+^ store depletion. The dysregulation of intracellular Ca^2+^ homeostasis partly explains the dysfunction of PASMCs in PAH; therefore, with SOCE being the crucial contributor to PASMCs Ca^2+^ homeostasis, Masson and collaborators deciphered its contribution to PAH pathogenesis and their potential therapeutic interests [7].

In addition to SOCE, PAECs, PASMCs, and pulmonary artery fibroblast expressed a variety of stretch-activated channels (SACs) activated by membrane stretch. The review by Solène Barbeau and colleagues updated the current knowledge of SAC in pulmonary circulation and its relevance in the physiopathology of PAH. SAC converts physical forces into biological signals and cell responses. SAC is a non-selective Ca^2+^-permeable cation channel, including proteins of the TRP (transient receptor potential) and Piezo channel superfamilies [8].

Potassium (K^+^) channels are critical for PAH pathogenesis because K^+^ channels are the primary determinants of vascular tone by regulating the resting membrane potential (Em) of cells. The Em of PASMCs and PAEC functions mainly control the pulmonary arterial tone. Resting membrane potential is dependent on membrane permeability in cations and anions. All mammalian cells, including PASMCs, have a negative Em close to the equilibrium potential for K^+^ ions (Ek). The electrical difference between the cytosol and the extracellular space ranges from −85 to −60 mV in excitable cells (including PASMC), close to the theoretical Ek (−90 mV). High intracellular K^+^ (140mM) and low extracellular K^+^ (5 mM) concentrations are mainly maintained by the Na^+^/K^+^ pump, while the opening of K^+^ channels hyperpolarizes the plasma membrane. Four major K^+^ channels found in the pulmonary vasculature are divided by their electrophysiological properties and structures: the two-pore domain channels (K2P), the ATP-sensitive potassium channel (KATP), a type of inwardly rectifying potassium channel (Kir), the voltage-gated potassium channels (Kv), and the Ca^2+^-activated K^+^ channels (KCa) [4]. 

All changes in K^+^ channel expression or function in PASMCs have potential repercussions on the plasma membrane resting membrane potential, PA tone, and PASMCs phenotype.

In their review, Carrie L. Welch and Wendy K. Chung updated recent advances in the current knowledge of gene variants of *ABCC8*, *ATP13A3*, and *KCNK3* in PAH, which have been validated in multiple PAH cohorts of PAH patients and which, to date, can explain ~2.7% of PAH cases. Identifying these ion channel mutations in *ABCC8*, APT13A3, and *KCNK3* suggests that these channels could be excellent targets for drugs in treating PAH [5].

To complement this review, Redel-Traub Gabriel and colleagues focused more on the pathophysiologic relevance of K^+^ channel dysfunction and the therapeutic potential of these K^+^ channels as drug targets in the context of PAH. The authors intensely discussed KCNK3, KATP, and Kv as promising therapeutic targets in PAH, with recent experimental pharmacologic discoveries significantly advancing the field [9].

In their study, Mohammed Al-Chawishly and coworkers used small-vessel myography to investigate the contribution of Kv7 channels. This K^+^ channel could be activated by cGMP or cAMP signaling to regulate rat pulmonary arterial tones. Using the pan-Kv7 blockers, linopirdine, and XE991, the authors found that Kv7 inhibition reduces PA relaxation induced by the NO donors or the riociguat sildenafil. Moreover, the authors demonstrated the protein expression of Kv7.1 and Kv7.4 proteins in rat pulmonary arteries, and that the selective activation of Kv7.1 and Kv7.4 channels, but not Kv7.5, caused PA relaxation. This study demonstrated that Kv7.4 channels contribute to endothelium-dependent PA relaxation by the cGMP-signaling pathway but have a lesser role in cAMP signaling [10].

In the study by Maria Callejo and collaborators, the authors demonstrated that severe experimental PAH rats induced by Sugen/hypoxia exposure suffered from vitamin D deficiency similarly to PAH patients. The authors compared the consequence of the restoration in vitamin D levels in pulmonary arterial tone, K^+^ channel function, and PAH phenotype. They found that recovering optimal vitamin D levels in severe PAH rats (Sugen/hypoxia) improved endothelial function and increased the function of KNCK3 K^+^ channels without improving right ventricular hemodynamics or pulmonary vascular remodeling, or right ventricle hypertrophy. These results suggest that vitamin D supplementation could benefit some pathophysiological features of PAH [11].

In their paper, Divya Guntur and colleagues revisited the role of the large-conductance Ca^2+^-activated K^+^ channels (BKCa) in pulmonary circulation. BKCa is vital due to its very high unitary conductance and ability to cause extreme changes in the membrane potential. This review updated the physiological mechanisms regulating BKCa open probability in pulmonary arterial vascular cells. Finally, this review identified the BKCa channel as a possible therapeutic target by regulating the pulmonary arterial tone and pulmonary vessel stiffness in various experimental animal models [12].

To conclude, this Special Issue of *Biomolecules* describes essential findings relating to the role of ion channels in PAH. All these data can be beneficial to furthering understanding of the complex PAH pathogenesis.
